# Supplementary material for: The low-density lipoprotein receptor promotes infection of multiple encephalitic alphaviruses
Source: Nat Commun. 2024 Jan 4;15:246. doi: 10.1038/s41467-023-44624-x (PMC10764363; doi:10.1038/s41467-023-44624-x)
Supplement: Supplementary file 1 — Supplementary Information [file 41467_2023_44624_MOESM1_ESM.pdf]

SUPPLEMENTARY FIGURES

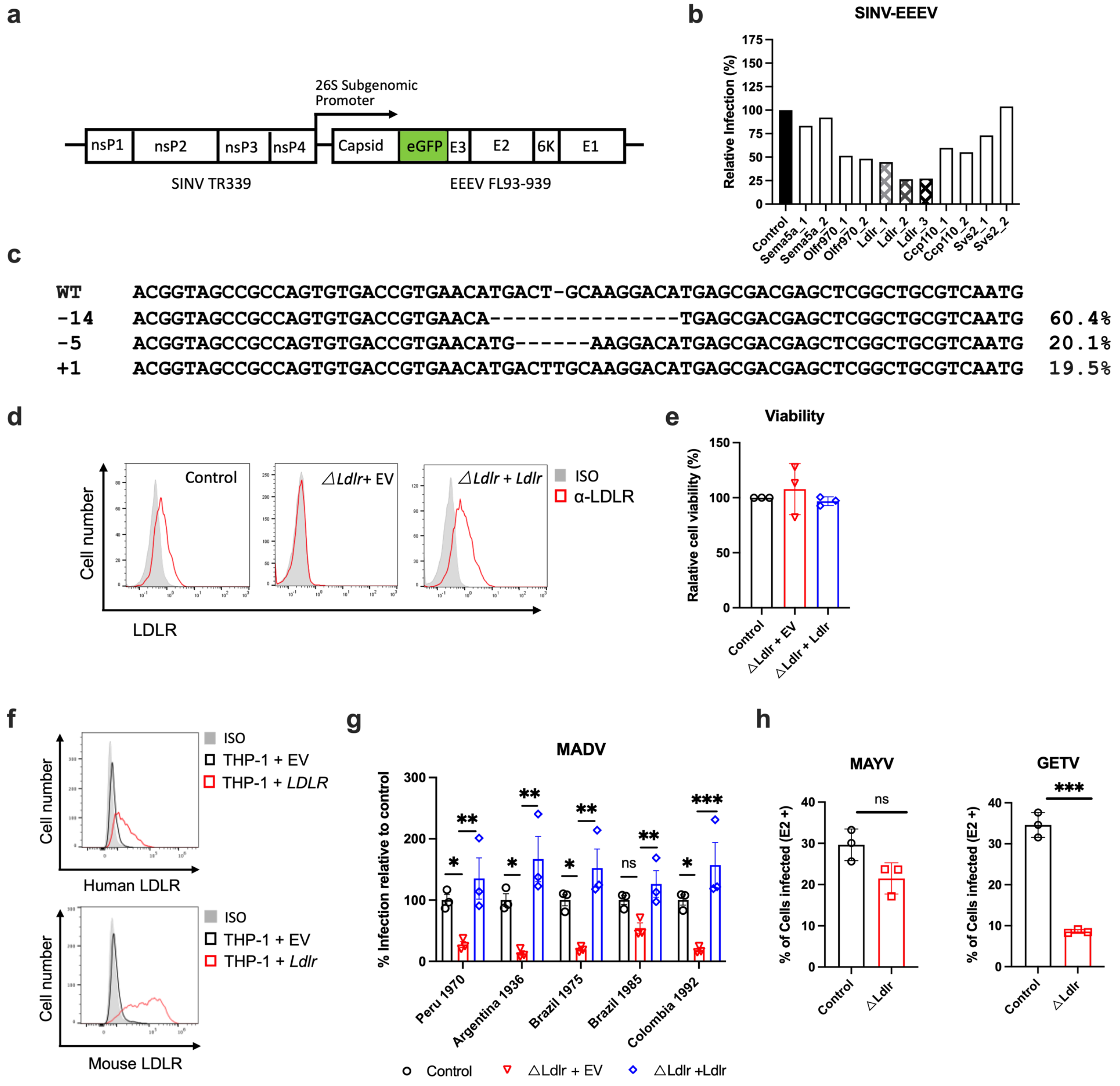

Supplementary Figure 1. LDLR expression promotes SINV-EEEV infection. a, Schematic of chimeric SINV-EEEV virus. The chimera contains the non-structural genes from SINV (strain TR339), structural genes from EEEV (FL93-939) and an *eGFP* reporter gene (green) between the capsid and E3 proteins. The insertion of GFP has minimal effects on virus infection and replication [44]. **b**, Validation of the five top plasma membrane gene hits by editing each gene with 2-3 independent sgRNAs in N2a cells. Cells were inoculated with SINV-EEEV, and infection was assessed by flow cytometry using GFP expression. **c**, Next-generation sequencing confirmation of *Ldlr* gene editing in N2a cells. Allele frequency is indicated next to each sequence. **d**, *Δ B4galt7* (control), *ΔB4galt7 ΔLdlr* (*ΔLdlr* + EV) and LDLR-complemented *ΔB4galt7 ΔLdlr* (*ΔLdlr* + *Ldlr*) N2a cells were analyzed for surface expression of LDLR by flow cytometry using an anti-LDLR

(red) or isotype control (grey) antibody. Representative histograms are shown. **e**, Viability of *ΔB4galt7* (control), *ΔB4galt7 ΔLdlr* (*ΔLdlr* + EV) and LDLR-complemented *ΔB4galt7 ΔLdlrad3* (*ΔLdlr* + *Ldlr*) N2a cells as determined by MTT assay (n = 3 experiments, mean ± SDs). **f**, Cell surface staining of LDLR expression in control THP-1 cells (THP-1 + EV), THP-1 cells expressing human LDLR (THP-1 + *LDLR, top*), and THP-1 cells expressing murine LDLR (THP-1 + *Ldlr*, bottom). Representative flow cytometry histograms are shown using an anti-LDLR (red or black) or isotype control (grey) antibody. **g**, *ΔB4galt7* control (Control), *ΔB4galt7 ΔLdlr* (*ΔLdlr* + EV), and LDLR-complemented *ΔB4galt7 ΔLdlr* (*ΔLdlr* + *Ldlr*) N2a cells were inoculated with different strains of MADV, and infection was assessed by staining with anti-E2 antibodies (n = 3 experiments, mean ± SDs). **h,** *ΔB4galt7* control and *ΔB4galt7 ΔLdlr* N2a cells were inoculated with MAYV and GETV. Infection was assessed by staining with anti-E2 antibodies (n = 3 experiments, mean ± SDs). Means ± SD are shown. Statistical analysis: (**g**) two-way ANOVA with Tukey test, (left-to-right) *P = 0.0174, **P = 0.0011, *P = 0.0194, **P = 0.0040, **P = 0.0028, *P = 0.0136, ***P = 0.0007; (**h**) two-tailed Student's t test, (left-to-right) *** P = 0.0001; ns, not significant. Source data are provided as a Source Data file.

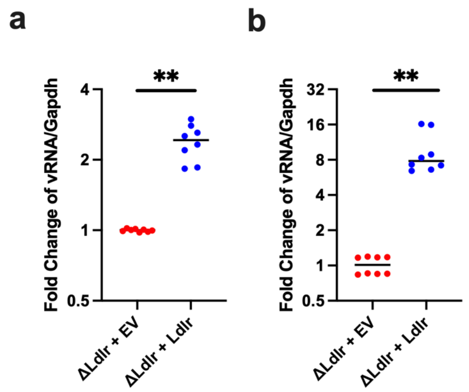

Supplementary Figure 2. LDLR expression promotes SINV-EEEV attachment and internalization in N2a cells. **a**, *ΔB4galt7 ΔLdlr* (*ΔLdlr* + EV), and LDLR-complemented *ΔB4galt7 ΔLdlr* (*ΔLdlr* + *Ldlr*) N2a cells were incubated with SINV-EEEV (FL93-939) at 4°C for 45 min. After serial washes, bound virions were quantified as the ratio of viral RNA (vRNA) to *Gapdh* mRNA levels via RT-qPCR and expressed as fold change compared to *ΔLdlr* + EV N2a cells. **b**, After removal of unbound virus, the temperature was increased to 37°C for 45 min to allow internalization. Intracellular viral RNA was measured and presented as in (**a**). Mean (n = 4 experiments in duplicate [all data shown]). Means ± SD are shown. Statistical analysis: two-tailed unpaired t test: (**a**) ***P* = 0.0100; (**b**) ***P* = 0.0030. Source data are provided as a Source Data file.

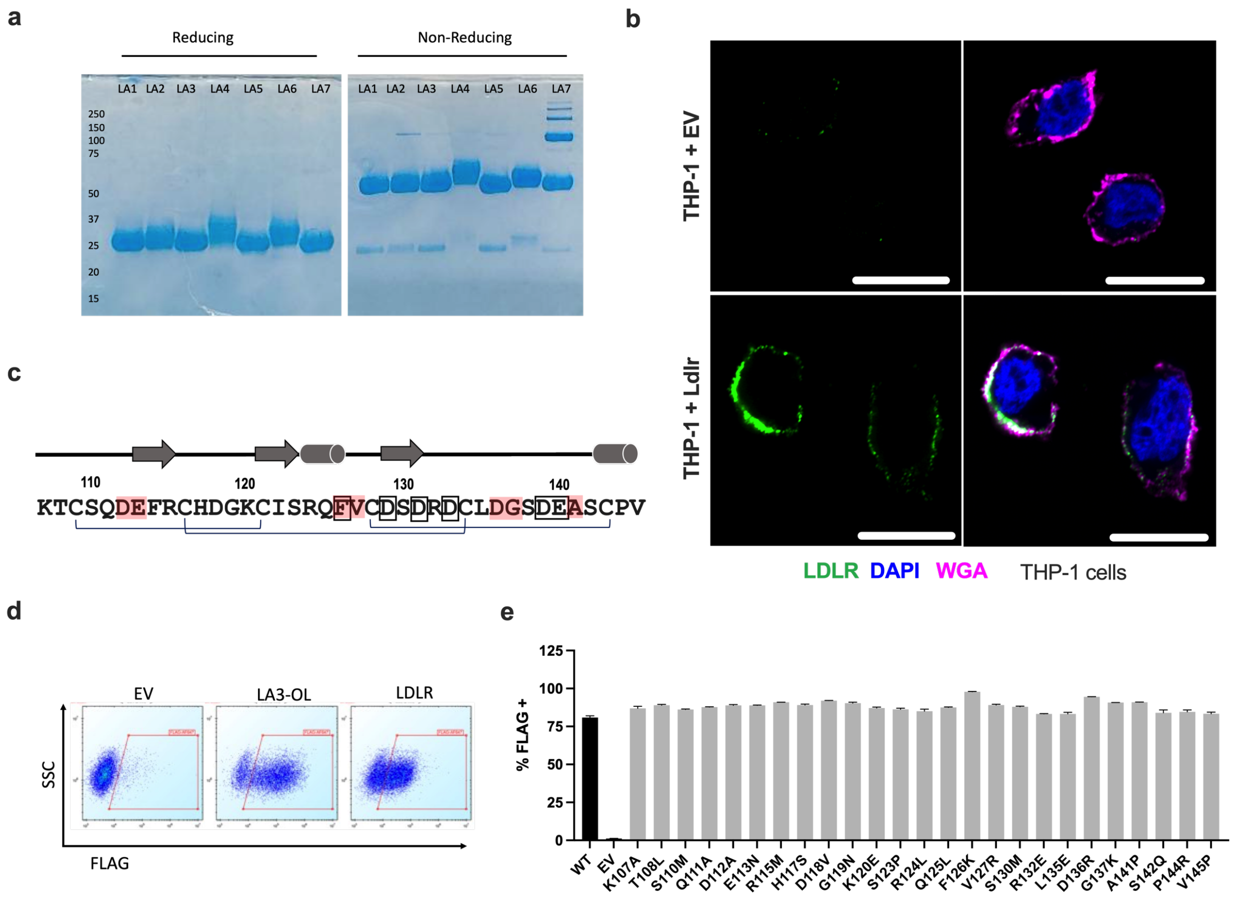

Supplementary Figure 3. **LDLR directly binds to EEEV virions.** **a**, Expression and characterization of recombinant LDLR LA proteins fused with human IgG Fc fragment. Reducing and non-reducing Coomassie-stained SDS–PAGE of human LDLR LA domain (LA1, LA2, LA3, LA4, LA5, LA6 and LA7) fused to human IgG1 Fc domain. Molecular weight markers are indicated (*left*). **b**, Staining of cell surface LDLR in THP-1 cells ectopically expressing murine LDLR (THP-1 + LDLR) or empty vector control (THP-1 + EV) with an anti-LDLR antibody. Confocal microscopy shows cell surface LDLR puncta (green), nuclei (DAPI, blue) and WGA (magenta, cell membrane). Scale bar, 10 µm. One representative image of 15 to 20 fields, n = 2 experiments. **c**, Schematic of LDLR LA3 domain secondary structure and position of key residues affecting SINV-EEEV infectivity. Disulfide bonds are indicated with brackets. Residues coordinating the calcium ion are framed. Residues important for SINV-EEEV infection are highlighted in salmon. **d**, Full-length human LDLR or mutated LA3 mini receptor expression on the surface of THP-1 cells as measured by flow cytometry an using anti-Flag-tag (N-terminal) antibody. One of three experiments is shown. **e**, Expression of indicated Flag-tagged LA3 mini receptors on the surface THP-1 cells. Expression was assessed by flow cytometry after N-terminal anti-Flag-tag staining. One representative of three experiments is shown. Source data are provided as a Source Data file.

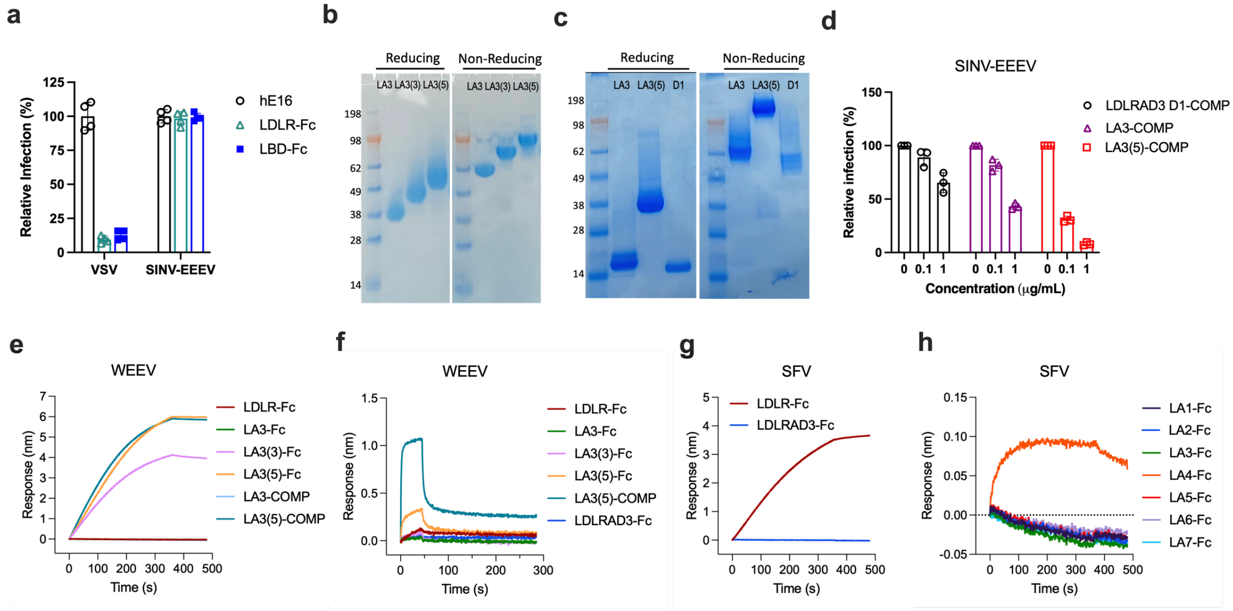

Supplementary Figure 4, **Neutralization of alphavirus infection by LA3 tandem repeat domain proteins. a**, Pre-incubation of SINV-EEEV or VSV with 10 μg/ml of control (hE16), LDLR-Fc, or LBD-Fc before infection of *ΔB4galt7* N2a cells (n = 3 experiments, mean ± SD). **b-c**, Reducing and non-reducing Coomassie-stained SDS–PAGE of recombinant LA concatemer proteins fused to human IgG Fc fragment (LA3-Fc; LA3(3)-Fc; LA3(5)-Fc) (**b**) or indicated LA domains pentamerized with COMP (LA3-COMP; LA3(5)-COMP; LDLRAD3 D1-COMP) (**c**) Molecular weight markers are indicated (*left*). **d**, Effects of pre-incubation of LA3-COMP, LA3(5)-COMP, or LDLRAD3 D1-COMP (negative control) proteins on SINV-EEEV infection in human SH-SY5Y neuronal cells (n = 3 experiments). **e-f**, Biolayer interferometry (BLI) graphs showing WEEV VLPs binding to immobilized LA3(5)-COMP, LA3(5)-Fc, and LA3(3)-Fc (**e**), WEEV VLPs binding to LA3(5)-COMP in solution (**f**), SFV VLPs binding to full length LDLR (LDLR-Fc) (**g**), and Fc-fused single LDLR LA domain (LA-Fc) (**h**) (one of two experiments is shown). Means ± SD are shown. Source data are provided as a Source Data file.

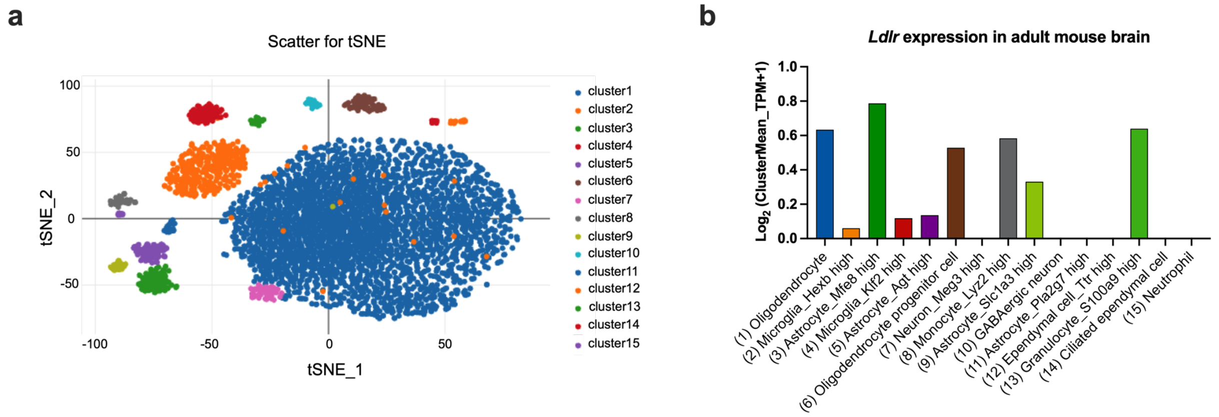

Supplementary Figure 5, ***Ldlr* expression profile in adult mouse brain**. **a-b**, Mouse Cell Atlas Database (https://bis.zju.edu.cn/MCA/index.html) search results for cell type clusters in the adult mouse brain represented as t-distributed Stochastic Neighbor Embedding (t-SNE) plots (**a**) and corresponding differential expression of *Ldlr* RNA in each cell cluster (**b**). Source data are provided as a Source Data file.

SUPPLEMENTARY DATA and TABLES

Supplementary Data 1. **CRISPR/Cas9 screen results**. See attached Excel file.

Supplementary Table 1. **MADV strains used**

Virus Name	Country	Collection Date	Source Of Isolate	Genbank Accession #
MADV	Argentina	1936	Horse	U01640, U01600, U01560
MADV	Peru	1970	Sentinel hamster (blood)	U01653, U01612, U01571
MADV	Brazil	1975	Mosquito	AF160174
MADV	Brazil	1985	Mosquito	AF159561
MADV	Colombia	1992	Sentinel hamster	AF160180

Supplementary Table 2. sgRNA used for validation.

Gene	sgRNA
Sema5a_1up	CTTCTTCCGAGAAAACGCCG
Sema5a_2up	AAGAACTTGTCGTAGGAGCG
Olfr970_1up	TAATAAAAGTATCAGAACAG
Olfr970_2up	CATAGATTGCTAGAAAGAGG
Ldlr_1up	AAAATGCATCGCTAGCAAGT
Ldlr_2up	GGTGTCGTAGGACAAGTTAG
Ldlr_3up	TGACCGTGAACATGACTGCA
Ccp110_1up	GCAATAACGTCACTGTTGAG
Ccp110_2up	GAAGAGGTCAAAGGAATACG
Svs2_1up	AGAGCATACAAGTGTCAAAG
Svs2_2up	TTCAGCCAACTAAAATCCCA
non-targeting control	GAAGTTCGAGGGCGACACCC
