# Supplementary material for: The low-density lipoprotein receptor promotes infection of multiple encephalitic alphaviruses
Source: Nat Commun. 2024 Jan 4;15:246. doi: 10.1038/s41467-023-44624-x (PMC10764363; doi:10.1038/s41467-023-44624-x)
Supplement: Supplementary file 3 — Description of Additional Supplementary Files [file 41467_2023_44624_MOESM3_ESM.docx]

**Description of Additional Supplementary Files**

**Supplementary Data 1: CRISPR/Cas9 screen results.** Positively selected genes in the screen were ranked according to their RRA (Robust Rank Aggregation) scores. These were determined by ranking sgRNAs based on their p-values calculated from the negative-binomial model and using a modified RRA algorithm named α-RRA to identify positively or negatively selected genes.
