# Supplementary material for: The low-density lipoprotein receptor promotes infection of multiple encephalitic alphaviruses
Source: Nat Commun. 2024 Jan 4;15:246. doi: 10.1038/s41467-023-44624-x (PMC10764363; doi:10.1038/s41467-023-44624-x)
Supplement: Supplementary file 5 — Reporting Summary [file 41467_2023_44624_MOESM5_ESM.pdf]

nature portfolio

Corresponding author(s): Michael Diamond

Last updated by author(s): Dec 15, 2023

Reporting Summary

Nature Portfolio wishes to improve the reproducibility of the work that we publish. This form provides structure for consistency and transparency in reporting. For further information on Nature Portfolio policies, see our Editorial Policies and the Editorial Policy Checklist.

Statistics

For all statistical analyses, confirm that the following items are present in the figure legend, table legend, main text, or Methods section.

n/a	Confirmed	
☐	☒	The exact sample size (n) for each experimental group/condition, given as a discrete number and unit of measurement
☐	☒	A statement on whether measurements were taken from distinct samples or whether the same sample was measured repeatedly
☐	☒	The statistical test(s) used AND whether they are one- or two-sided<br>*Only common tests should be described solely by name; describe more complex techniques in the Methods section.*
☒	☐	A description of all covariates tested
☐	☒	A description of any assumptions or corrections, such as tests of normality and adjustment for multiple comparisons
☐	☒	A full description of the statistical parameters including central tendency (e.g. means) or other basic estimates (e.g. regression coefficient) AND variation (e.g. standard deviation) or associated estimates of uncertainty (e.g. confidence intervals)
☐	☒	For null hypothesis testing, the test statistic (e.g. F, t, r) with confidence intervals, effect sizes, degrees of freedom and P value noted<br>*Give P values as exact values whenever suitable.*
☒	☐	For Bayesian analysis, information on the choice of priors and Markov chain Monte Carlo settings
☒	☐	For hierarchical and complex designs, identification of the appropriate level for tests and full reporting of outcomes
☒	☐	Estimates of effect sizes (e.g. Cohen's d, Pearson's r), indicating how they were calculated

Our web collection on statistics for biologists contains articles on many of the points above.

Software and code

Policy information about availability of computer code

Data collection	No software was used in this study to collect data
Data analysis	Prism 8.0 was used to perform most data analysis. Raw BLI response traces were processed using GatorOne Software v2.7. All flow cytometry data were processed using FlowJo 10.0. Luminescence was read on a Synergy H1 Hybrid Multi-Mode Reader (BioTek). The sgRNA sequences against specific genes were obtained after removal of the tag sequences using the FASTX-Toolkit (http://hannonlab.cshl.edu/fastx_toolkit/) and cutadapt (version 1.8.1). Confocal microscopy data were processed and analyzed with ImageJ software v.1.53t.

For manuscripts utilizing custom algorithms or software that are central to the research but not yet described in published literature, software must be made available to editors and reviewers. We strongly encourage code deposition in a community repository (e.g. GitHub). See the Nature Portfolio guidelines for submitting code & software for further information.

Data

Policy information about availability of data

All manuscripts must include a data availability statement. This statement should provide the following information, where applicable:

- Accession codes, unique identifiers, or web links for publicly available datasets
- A description of any restrictions on data availability
- For clinical datasets or third party data, please ensure that the statement adheres to our policy

All data supporting the findings of this study are available within the main text and supplemental data. Source data for main and supplemental figures are provided

with this paper. All reagents will be made available on request after completion of a Materials Transfer Agreement.

Research involving human participants, their data, or biological material

Policy information about studies with human participants or human data. See also policy information about sex, gender (identity/presentation), and sexual orientation and race, ethnicity and racism.

Reporting on sex and gender	N/A
Reporting on race, ethnicity, or other socially relevant groupings	N/A
Population characteristics	N/A
Recruitment	N/A
Ethics oversight	N/A

Note that full information on the approval of the study protocol must also be provided in the manuscript.

Field-specific reporting

Please select the one below that is the best fit for your research. If you are not sure, read the appropriate sections before making your selection.

☒ Life sciences ☐ Behavioural & social sciences ☐ Ecological, evolutionary & environmental sciences

For a reference copy of the document with all sections, see nature.com/documents/nr-reporting-summary-flat.pdf

Life sciences study design

All studies must disclose on these points even when the disclosure is negative.

Sample size	No sample sizes were chosen a priori but instead estimated based on prior knowledge of anticipated experimental differences among groups. All experiments with statistical analysis were repeated at least two independent times, each with multiple technical replicates. Experimental size of animal cohorts was determined based on prior experience performing studies in mice.
Data exclusions	No data was excluded.
Replication	All experiments had multiple biological and/or technical replicates and are indicated the Figure legends.
Randomization	For animal studies, mice were randomly assigned from large batches obtained from the vendor to treatment groups in an age-matched distribution.
Blinding	No blinding was performed although several key studies were performed independently by multiple members of the group

Reporting for specific materials, systems and methods

We require information from authors about some types of materials, experimental systems and methods used in many studies. Here, indicate whether each material, system or method listed is relevant to your study. If you are not sure if a list item applies to your research, read the appropriate section before selecting a response.

Materials & experimental systems

n/a	Involved in the study	
☐	☒	Antibodies
☐	☒	Eukaryotic cell lines
☒	☐	Palaeontology and archaeology
☐	☒	Animals and other organisms
☒	☐	Clinical data
☒	☐	Dual use research of concern
☒	☐	Plants

Methods

n/a	Involved in the study	
☒	☐	ChIP-seq
☐	☒	Flow cytometry
☒	☐	MRI-based neuroimaging

Antibodies

Antibodies used	Complemented cells were assessed for LDLR surface expression using an anti-Flag antibody (Cell Signaling Technology, 14793) and Alexa Fluor 647-conjugated goat anti-rabbit IgG (Thermo Fisher, A27040) or anti-LDLR antibody (Thermo Fisher, PA5-46987, 1:200)

and Alexa Fluor 647-conjugated secondary antibody (Thermo Fisher, A-11055, 1:1000).

Cells infected with MADV viruses were stained with DC2.112 mAb (1:1,000 dilution), followed by goat-anti-human IgG (Thermo Fisher, A21445, 1:1,000).

To test LDLR or its LA domains for binding to EEEV VLPs, plates were coated with anti-EEEV mAb EEEV-10 (generated in Diamond laboratory) at 1 µg/mL overnight

Validation	All primary Abs were validated using purified viral proteins by ELISA or transfected/infected cells by flow cytometry. All secondary antibodies were validated against indicated proteins by the manufacturer per their associated Data Sheets.

Eukaryotic cell lines

Policy information about cell lines and Sex and Gender in Research

Cell line source(s)	Neuro 2a (N2a, ATCC CCL-131), HEK 293T (ATCC CRL-3216) and THP-1 cells (ATCC TIB-202) were obtained commercially. ΔB4galt7 N2a cells were generated in the Diamond laboratory. For genetically modified N2a cells (LDLR KO or complemented cells, generated in the Diamond laboratory), selection was maintained using the following antibiotics: puromycin (2.5 µg/mL, InvivoGen), blasticidin (4 µg/mL, InvivoGen) or hygromycin (200 µg/mL, InvivoGen).
Authentication	These cells grew as expected and propagated virus as expected. Also, we confirmed expression of specific transgenes using antibodies and flow cytometry.
Mycoplasma contamination	All cell lines are routinely tested each month and were negative for mycoplasma.
Commonly misidentified lines (See ICLAC register)	This study did not involve any commonly misidentified cell lines.

Animals and other research organisms

Policy information about studies involving animals; ARRIVE guidelines recommended for reporting animal research, and Sex and Gender in Research

Laboratory animals	Specific-pathogen-free C57BL/6J mice were purchased from Jackson Laboratories (Cat #000664) and maintained in a specific-pathogen-free facility. Four-week-old male mice were used in all experiments. Experimental procedures were approved by the Washington University School of Medicine Institutional Animal Care and Use Committee (assurance number A3381-01) and followed guidelines of the Guide for the Care and Use of Laboratory Animals. Mice were housed in groups of 3 to 5. Photoperiod = 12 hr on:12 hr off dark/light cycle. Ambient animal room temperature is 70° F, controlled within ±2° and room humidity is 50%, controlled within ±5%.
Wild animals	No wild animals were used in this study.
Reporting on sex	Only male mice were used in this study since the virological and clinical phenotypes after MADV infection are more consistent in our experience.
Field-collected samples	No field collected samples were used in this study.
Ethics oversight	All experiments were conducted with approval of the Institutional Animal Care and Use Committee at the Washington University School of Medicine (Assurance number A3381-01)

Note that full information on the approval of the study protocol must also be provided in the manuscript.

Plants

Seed stocks	N/A
Novel plant genotypes	N/A
Authentication	N/A

Flow Cytometry

Plots

Confirm that:

☒ The axis labels state the marker and fluorochrome used (e.g. CD4-FITC).

☒ The axis scales are clearly visible. Include numbers along axes only for bottom left plot of group (a 'group' is an analysis of identical markers).

☒ All plots are contour plots with outliers or pseudocolor plots.

☐ A numerical value for number of cells or percentage (with statistics) is provided.

Methodology

Sample preparation	For single- (< 10 h) or multi- (< 24 h) step infection assays, ΔB4galt7, ΔB4galt7 ΔLdlr, and Ldlr-complemented N2a cells were inoculated with the following viruses: SINV-EEEV (FL93-939) (MOI of 100, 8.5 h or MOI of 10, 24 h), MADV (Peru 1970, MOI of 10, 8 h; Argentina 1936, MOI of 10, 8 h, Brazil 1975, MOI of 20, 8 h; Brazil 1985 MOI of 10, 8 h, Colombia 1992, MOI pf 20, 8 h), SINV-VEEV (TrD) (MOI of 20, 7.5 h), SINV-WEEV (CBA87) (MOI of 10, 18.5 h), SINV (TR339) (MOI of 1, 24 h), SINV-CHIKV (MOI of 10, 7.5 h), SINV-SFV (MOI of 0.3, 16 h), MAYV (MOI of 3, 18 h), and GETV (MOI of 1, 18 h). LDLR-expressing THP-1 cells were inoculated with SINV-EEEV (MOI of 10, 16 h), SINV-WEEV (MOI of 10, 24 h), or SINV-SFV (MOI of 10, 24 h), respectively. At indicated time points, cells were collected using trypsin and fixed with 1% or 2% paraformaldehyde (PFA) in PBS for 15 min at room temperature.
Instrument	MACSQuant Analyzer 10 (Miltenyi Biotec) or an iQue3 Cytometer (Sartorius)
Software	FlowJo 10.0
Cell population abundance	No sorting was performed.
Gating strategy	Cells were assessed by FSC/SSC and live/dead staining and then evaluated for GFP expression or antibody staining. Uninfected cells and isotype control antibodies were used to set negative gates depending on the experiment.

☐ Tick this box to confirm that a figure exemplifying the gating strategy is provided in the Supplementary Information.
